# Patient Navigation Behavior in a Symptom-Based Self-Triage Mobile App for Direct-to-Consumer Urgent Care: Retrospective Observational Study

**DOI:** 10.2196/63816

**Published:** 2025-11-04

**Authors:** Tarso Augusto Duenhas Accorsi, Flavio Tocci Moreira, Anderson Aires Eduardo, Renata Albaladejo Morbeck, Karen Francine Köhler, Karine De Amicis Lima, Carlos Henrique Sartorato Pedrotti

**Affiliations:** 1 Telemedicine Department Hospital Israelita Albert Einstein São Paulo Brazil; 2 Digital Platform Hospital Israelita Albert Einstein São Paulo Brazil

**Keywords:** telemedicine, mobile health, mHealth, mobile apps, self-triage, symptom checker, user behavior, patient navigation, human-computer interaction, urgent care, digital health

## Abstract

**Background:**

Patient interaction patterns with self-triage modules in mobile health apps during urgent direct-to-consumer telemedicine consultations remain underexplored, despite their critical role in optimizing virtual care pathways.

**Objective:**

This study aimed to analyze user navigation behaviors within the screen pathways of a symptom-based self-triage mobile app’s algorithm during remote urgent care assessments.

**Methods:**

This observational, retrospective, single-center study analyzed data from users who were aged 18 years and older and who voluntarily sought virtual urgent care through the Einstein Conecta (version 2.0; iOS and Android) at a private Brazilian hospital between May 2022 and December 2023. Patients with incomplete connection records were excluded. User interactions were evaluated based on the number of distinct triage flows accessed per session, the number of screens viewed per flow, the frequency of returns to previous screens, and the time spent within the self-triage module. Descriptive statistical methods were applied for analysis.

**Results:**

Data from 62,006 unique users with a mean age of 36.51 (SD 10.53) years, of whom 54.65% (33,889/62,006) were female, were analyzed. They initiated 194,976 self-triage flows. We found that 36.89% (22,875/62,006) of users completed 1 flow per session; 22.15% (13,734/62,006) users accessed 2 flows; and 27.93% (17,317/62,006) users accessed ≥4 flows (maximum 63 flows). Users receiving an initial emergency department referral recommendation were more likely to initiate subsequent flows than those recommended for virtual assessment. Returning to a previous screen was infrequent (used by 5277/62,006, 8% of users). The average time spent in the first flow was 70.95 (SD 65.26) seconds, with an average of 9.51 (SD 12.84) seconds per screen.

**Conclusions:**

In this cohort, most users explored alternative pathways beyond the initial self-triage recommendation, particularly when directed to the emergency department, while rarely backtracking within a flow. These findings underscore the need to refine self-triage mechanisms in telemedicine to better align with observed user navigation behaviors and preferences.

## Introduction

### Background

Direct-to-consumer (DTC) telemedicine serves as an increasingly common initial point of contact with the health care system for patients experiencing acute conditions [1]. Within this landscape, mobile health (mHealth) apps have become a primary interface, largely due to their perceived accessibility and ease of use [2]. A frequent initial step within these virtual urgent care pathways is a self-triage process, where patients interact with an app, often driven by symptom checker algorithms, to determine the appropriate level and immediacy of care needed [3,4]. These systems typically rely on evidence-based questioning to guide users.

The fundamental goal of self-triage in urgent care contexts is to facilitate appropriate care navigation, particularly ensuring timely referral to an in-person emergency department (ED) for evaluation and treatment of potentially serious conditions [5]. Consequently, symptom checker algorithms often adopt a conservative approach to minimize risk, although achieving consistently high accuracy in both triage recommendations and diagnostic precision remains a challenge [6].

Beyond algorithmic accuracy, the effectiveness of self-triage is significantly influenced by user behavior and engagement with the digital interface, aspects that remain insufficiently understood. How patients navigate triage questions, choose alternative pathways, or reconsider their responses can provide valuable insights into the usability and perceived utility of these apps [7]. Factors such as the perceived predictability of the digital environment and the user’s ability to meet their immediate health care goals can shape decision-making during self-triage. Furthermore, individual circumstances, including perceived condition severity, access barriers to physical facilities, health insurance constraints, or employment concerns, may influence how users interact with self-triage recommendations. Understanding these interaction patterns—such as the frequency with which users explore multiple symptom pathways (“flows”) or navigate back within a flow—is crucial for optimizing system design and ensuring alignment with patient needs and expectations. Deviations from a single, linear triage pathway may indicate user uncertainty, exploration of options, or potential dissatisfaction with the presented care recommendations [8,9].

Previous evaluations of digital symptom checker tools have examined their diagnostic accuracy and use in various settings [10,11]. However, detailed analyses of how users navigate these triage systems—such as switching between symptom flows or revisiting previous questions—remain limited, especially in large real-world cohorts [12,13]. In particular, user navigation habits in telemedicine self-triage may differ from those in general health apps or nonmedical apps due to the urgency and high stakes of acute care decisions. Understanding where our approach fits, we build on these studies by focusing specifically on navigation behavior within a telemedicine, symptom-based triage app, an aspect that has not been thoroughly explored in previous research.

The Einstein Conecta (version 2.0) platform has previously been implemented in urgent care telemedicine, with a study demonstrating its effectiveness in risk stratification. In a large retrospective study of more than 230,000 telemedicine consultations, our team evaluated ED referral patterns and adherence to red-flag guidelines in a DTC setting using Einstein Conecta. Only 6.05% of patients were referred to the ED, with 98.6% of these referrals aligned with institutional protocols. Even when no guideline was explicitly applied, 97.6% of referrals were considered clinically justified, highlighting the safety and consistency of protocol-driven virtual care [14].

Despite the growing adoption of mHealth self-triage, detailed knowledge about patient interaction patterns within these specific app interfaces, particularly concerning the navigation across different screens and flows during urgent care seeking, is limited. We hypothesized that patient adherence to specific self-triage pathways might be influenced by the nature of the algorithmic guidance, particularly when encountering recommendations for in-person ED visits versus continued virtual care. Therefore, this study aimed to analyze user navigation behaviors within the screen pathways of a symptom-based self-triage mHealth app during DTC telemedicine urgent care consultations, focusing on metrics such as the number of distinct triage flows accessed, the number of screens viewed per flow, the frequency of returning to previous screens, and the time spent interacting with the app.

### Related Work

Digital symptom checkers have become popular as DTC triage tools in telemedicine, leading to extensive assessments of their accuracy, safety, and usability. These apps generally use risk-averse algorithms—favoring sensitivity over specificity—to prevent missing emergencies, often resulting in overtriage. Notably, systematic reviews have found no evidence that this cautious approach harms patient safety [15]. Research in acute care settings indicates that artificial intelligence–driven self-triage can accurately reflect professional assessments. For instance, a prospective trial in an ED showed that a symptom checker’s urgency advice matched nurse triage decisions in approximately 95% of cases [16]. Large-scale deployments suggest widespread public acceptance. Morse et al [17] reported more than 26,000 self-assessments through a symptom checker within 9 months at a US health system, with use focused more on younger adults (with an average age of approximately 34.3, SD 14.4 years) and female users (67%), and nearly half of the assessments took place outside clinic hours. Such tools have been incorporated into urgent care pathways, and their triage recommendations generally align with those of clinicians or nurse advice lines in identifying suitable care levels.

From a usability and human-computer interaction perspective, user feedback is generally positive. Patients often find symptom checkers easy to use and helpful for decision-making, reporting higher confidence and less anxiety after using them. Arellano Carmona et al [18] surveyed 2437 symptom checker users and found high levels of perceived usefulness and understanding, with many users feeling “empowered to seek medical help” after using the app. Consistently, early surveys and systematic reviews reported high satisfaction among symptom checker users [18]. Nevertheless, qualitative findings reveal areas for improvement. In one study, young adults described web search engines as faster and more flexible for symptom research, while symptom checkers were appreciated for personalized guidance but criticized for medical jargon and accuracy issues. Many participants were initially unaware that such digital triage tools existed, highlighting awareness and design gaps that can influence navigation habits in mHealth platforms [19].

Despite the increasing research on digital symptom checkers, important gaps remain in understanding how real users navigate these tools, especially how they interact with multistep or multiflow pathways in self-triage apps. Most previous studies have centered on single-use scenarios, often using standardized vignettes or controlled simulations, rather than observing how users naturally and repeatedly engage over time. As a result, there is limited knowledge about how users interact with these apps in an iterative way—for instance, whether they restart or explore different symptom flows if they are dissatisfied with initial results, or how they switch between various features within urgent care apps. Few studies explore why and how consumers actually use symptom checkers in practice, and the literature has consistently emphasized the need for more research into real-world use and decision-making processes. Addressing this gap is essential: understanding multiflow navigation and actual decision patterns can uncover usability issues and guide better design improvements that are not visible in single-flow studies.

### This Study

This study adds to this emerging field by examining user behavior across multiple consultation flows within a live urgent care self-triage app, providing new insights into how patients navigate and make decisions in a DTC mHealth urgent care setting. Focusing on real-world, multiflow use broadens the current understanding and helps fill an important gap regarding user experience and decision pathways in digital self-triage systems.

## Methods

### Study Design and Participants

This observational, retrospective, single-center study was conducted at the Telemedicine Center of Hospital Israelita Albert Einstein (HIAE) in São Paulo, Brazil. HIAE is a private, tertiary care hospital providing a comprehensive range of medical services. Access to its Telemedicine Center, including the virtual urgent care service analyzed in this study, typically requires private payment or coverage by specific health insurance plans. All necessary data were accessed through the institution’s secure digital records system. Data collection and confidential storage were managed by telemedicine physicians, while the Telemedicine Center team conducted data analysis and oversaw the study. All authors collaboratively wrote the manuscript, approved the final version, and decided to submit it for publication, certifying the data’s integrity and adherence to the protocol.

The study encompassed a population of patients aged 18 years and above who voluntarily approached the virtual ED for care between May 2022 and December 2023. The inclusion criteria were broad, allowing patients presenting with any acute condition to be part of the study. The sole exclusion criterion was the occurrence of connection issues that precluded the creation of medical records. Such instances led to the exclusion of participants from the study, as their medical evaluations could not be completed, leaving no trace of their visits in the database.

Patients presenting with any acute condition were eligible. Recruitment occurred passively and the study population consisted of a convenience sample of consecutive users who chose to access the virtual ED service via the app during the study period. Patients typically become aware of the service through hospital communications, health insurance provider information, or previous engagement with HIAE.

### The Einstein Conecta App and Self-Triage Algorithm

The Einstein Conecta app, available for both Android and iOS platforms, served as the interface for accessing the virtual urgent care service. Before initiating the self-triage process, users were typically required to log in or register, which linked their session to their institutional medical record and verified their insurance and payment status.

**Figure 1:**
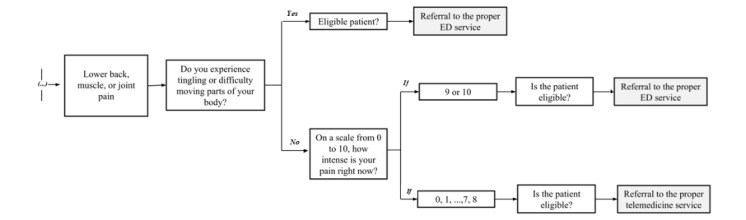
Self-triage algorithm for lower back, muscle, or joint pain, depicting the decision tree structure underlying the Einstein Conecta (version 2.0) app. ED: emergency department.

**Figure 2:**
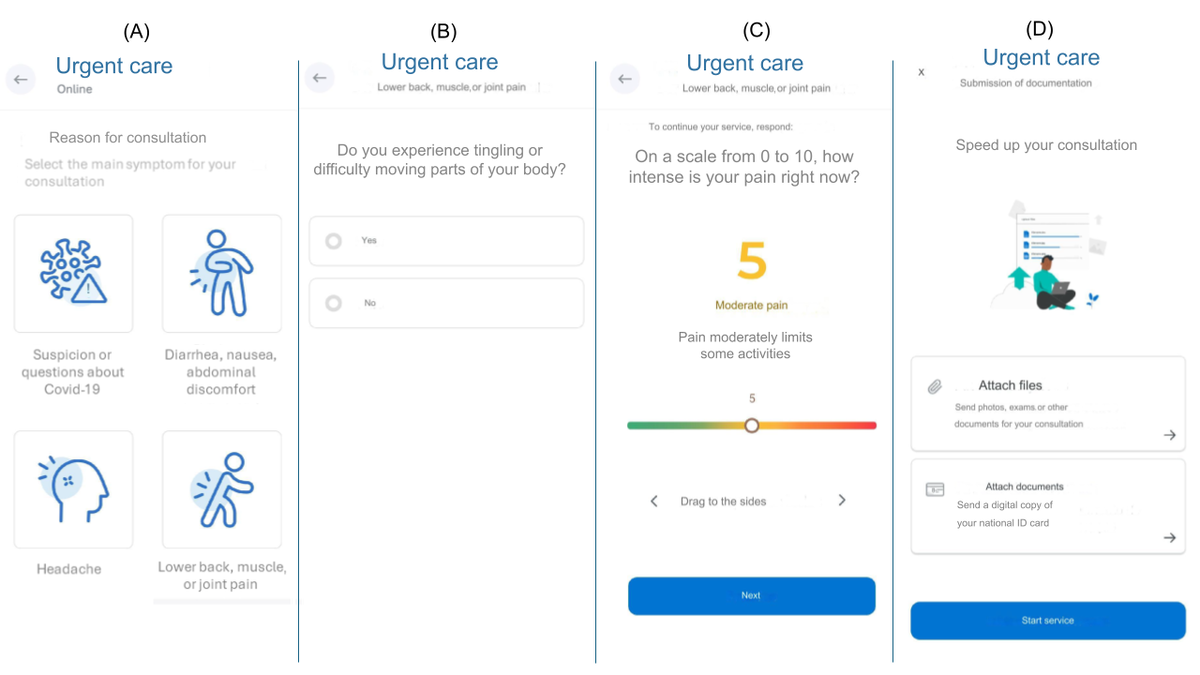
Self-triage app layout example assessed by smartphone. (A) The app’s home page requests the reason for the consultation. This image shows COVID-19; diarrhea; nausea; abdominal discomfort; headache; and lower back, muscle, or joint pain. (B) A question regarding the condition related to lower back, muscle, or joint pain: “Do you have tingling or difficulty moving part of your body?” (C) A question about the intensity of the pain at the moment. (D) Submission of documentation for the consultation.

Upon initiating a virtual urgent care consultation, patients first interacted with a self-triage module. This module featured an algorithm structured as a logical decision tree, designed and regularly updated by a dedicated team of IT and telemedicine experts based on current medical evidence and guidelines adapted for virtual assessment. The self-triage system included 17 distinct initial pathways or “flows,” corresponding to common urgent care concerns (eg, respiratory symptoms, abdominal pain, headache, etc). Each flow consisted of a sequence of questions designed to identify symptoms, assess severity, and screen for “red flags” indicative of potentially serious conditions. An example of a decision tree structure for 1 flow (lower back, muscle, or joint pain) is shown in Figure 1, and example user interface screens are shown in Figure 2.

On the basis of the user’s responses, each flow ultimately guided the patient toward one of two main recommendations: (1) referral for an immediate in-person evaluation at an ED or (2) placement in a queue for a virtual medical consultation via telemedicine. The algorithms were intentionally designed with high sensitivity for red flags to prioritize safety. While the system did not automatically dispatch emergency services, it provided clear directives for seeking in-person care when necessary [14].

The app allowed users flexibility during the self-triage process. Patients could navigate away from their initially selected symptom flow and choose an alternative flow if they felt it better represented their condition. Users could also navigate back to a previous question or screen within the same flow to change their response. The interface was designed to be intuitive, considering the urgency of situations and diverse user backgrounds. The system primarily focused on common acute physical concerns. Dedicated algorithms for complex chronic conditions or primary mental health crises were not part of the 17 core urgent care flows during the study period, although related symptoms might have led to an ED referral. A previous study using data from this app has examined related aspects of the telemedicine service [5].

### Outcome Measures

The primary goal was to analyze user interaction patterns within the self-triage module. Key metrics (primary outcomes) extracted for analysis are shown in Textbox 1.

**Key metrics (primary outcomes).**

- Number of distinct self-triage flows accessed per user session: this metric indicated whether users adhered to a single pathway or explored multiple symptom-based flows during 1 engagement with the virtual urgent care access point. Exploring multiple flows suggested user uncertainty, changing symptoms, or searching for a different outcome or recommendation.
- Number of screens accessed within each algorithm flow: this reflected how deeply users progressed within a specific flow before reaching a recommendation or switching to another flow.
- Number of returns to a previous screen within the same flow: this metric captured instances where users reconsidered or changed their answer to a preceding question. Frequent returns indicated confusion or attempts to alter the pathway outcome.
- Total time spent in the app’s self-triage algorithm on first access: this measured the duration from initiating self-triage to receiving the first recommendation (emergency department referral or queue for telemedicine).
- Average time spent per screen during the first access: this calculated the average time users spent on each question or information screen within the self-triage module. These time metrics related to the efficiency and potential usability of the interface.
- Total time spent in the app’s self-triage algorithm during reaccesses (if applicable): this measured the duration for subsequent flows accessed within the same user session.

### Data Extraction

Self-triage interaction data were extracted directly from the secure, centralized transactional database logs of the Einstein Conecta app system. User sessions were identified using unique patient identification codes linked to their institutional record. Timestamps associated with screen views, flow initiation, and flow completion (selection of a final recommendation) allowed for the reconstruction of navigation pathways and duration calculations. These interaction data were then linked to the corresponding telemedicine consultation records (if applicable) stored in the institutional database to form a comprehensive dataset for analysis.

### Statistical Analysis

Statistical analyses were performed using IBM SPSS Statistics for Windows (version 22.0; IBM Corp). The analysis used descriptive statistics. Categorical variables, such as the number of flows accessed or screens viewed, are presented as counts and percentages. Continuous variables, such as time spent, are presented as means and SDs. No missing data were encountered for the primary interaction variables analyzed. Given the exploratory nature of the study focused on describing interaction patterns, inferential statistics were not the primary focus.

### Ethical Considerations

The study protocol, designated “Tele Connect study,” received approval from the Institutional Ethics Committee of Hospital Israelita Albert Einstein (Certificado de Apresentação de Apreciação Ética—CAAE: 74197023.2.0000.0071). As the study was based on the retrospective analysis of anonymized data derived from routine care, the requirement for obtaining informed consent was formally waived by the committee. All procedures complied with national and international ethical standards, including the principles outlined in the Declaration of Helsinki.

## Results

### Participant Characteristics and Overall Triage Outcomes

Data from 62,006 unique patient users who accessed the Telemedicine Center via the Einstein Conecta app between May 2022 and December 2023 were analyzed. The mean age of users was 36.51 (SD 10.53) years, and 33,889 (54.65%) were female. Other sociodemographic data were limited in this dataset.

During the study period, these users initiated 194,976 self-triage flows through the app. Of these completed flows, 80,451 (41.26%) resulted in a recommendation for a virtual assessment via telemedicine, while the remaining 114,525 (58.74%) resulted in a recommendation to seek immediate in-person evaluation at an ED. Among the patients who were initially recommended for and proceeded to a virtual assessment, 13.73% (11,044/80,451) were subsequently referred for an in-person ED evaluation by the telemedicine physician during the consultation.

### User Interaction With Triage Flows

Analysis of user navigation patterns revealed variations in adherence to a single triage flow per session. Of the 62,006 users, 22,875 (36.89%) completed only 1 self-triage flow during their session. A significant portion of users accessed multiple flows: 13,734 (22.15%) accessed 2 distinct flows; 8080 (13.03%) accessed 3 flows; and the remaining 17,317 (27.93%) users accessed 4 or more flows. The maximum number of distinct flows accessed by a single user in 1 session was observed to be 63. While each initial concern corresponded to a specific primary flow, users could revisit or initiate different flows as needed within a session. Table 1 provides a detailed breakdown of the number of flows accessed per user.

**Table 1:** App algorithm flow accessed by each user (N=62,006).

App flow accessed	Users, n (%)
1	22,875 (36.89)
2	13,734 (22.15)
3	8080 (13.03)
4	5135 (8.28)
5	3436 (5.54)
6	2320 (3.74)
7	1595 (2.57)
8	1090 (1.76)
9	863 (1.39)
≥10	2878 (4.64)

### Screen Navigation Within Flows

Within each initiated flow (n=194,976), the number of screens viewed by the user varied. The algorithms for different chief concerns had varying lengths, ranging from 1 to 11 screens. Most flow interactions involved viewing only 1 screen (n=89,998, 46.16%) or 2 screens (n=85,402, 43.80%) before a recommendation was reached or the user navigated elsewhere. Deeper navigation within a flow was less common, with only a small fraction of interactions proceeding beyond 3 screens (Table 2).

**Table 2:** Screens accessed within each algorithm flow (n=194,976).

Screens per flow accessed	Algorithm flow, n (%)
1	89,998 (46.15)
2	85,402 (43.80)
3	18,268 (9.36)
4	1076 (0.55)
5	176 (0.09)
6	37 (0.02)
7	7 (0)
8	6 (0)
9	1 (0)
10	3 (0)
11	2 (0)

### Use of Back Navigation

Returning to a previous screen within the same flow was infrequent. Most (56,638/62,006, 91.34%) users did not use the back navigation feature at all during their triage process. A small percentage of users (n=4245, 6.85%) returned to a previous screen once, and fewer still (n=1123, 1.81%) returned multiple times (Table 3).

**Table 3:** Returns to a previous screen in a flow (N=62,006).

Returns to a previous screen in the same flow	Users, n (%)
0	56,638 (91.34)
1	4245 (6.84)
2	819 (1.32)
3	213 (0.34)
4	50 (0.08)
5	23 (0.04)
6	10 (0.02)
≥7	8 (0.01)

### Time Spent in Self-Triage

Regarding the duration of interaction, the average time users spent in the self-triage module during their *first accessed flow* within a session was 70.95 (SD 65.26) seconds. The average time spent viewing each screen within the self-triage module was approximately 9.51 (SD 12.84) seconds, calculated across all accessed screens in the first flow.

### Flow Switching Based on Initial Recommendation (Planned Analysis)

To explore potential reasons for accessing multiple flows, we descriptively analyzed whether the recommendation from the *first completed flow* influenced the likelihood of initiating a *second* flow within the same session. Among users who completed an initial flow resulting in an ED referral recommendation, 66% (75,587/114,525) initiated at least 1 subsequent flow. In contrast, among users whose first flow resulted in a recommendation for a virtual telemedicine assessment, 9% (7241/80,451) initiated a subsequent flow. This suggests that receiving an ED referral from the self-triage was associated with a higher tendency for users in this cohort to seek alternative pathways within the app compared to receiving a telemedicine recommendation.

## Discussion

### Principal Findings

This study analyzed user interaction patterns within a mobile self-triage app used for accessing DTC urgent care telemedicine. Our primary findings indicate that while the app facilitated rapid interaction (average time per screen approximately 9.5 seconds and average first flow duration approximately 71 seconds), only about one-third (22,875/62,006, 36.9%) of users completed just a single triage flow per session. The majority explored multiple flows, with users receiving an initial recommendation for an in-person ED visit being relatively more likely to initiate subsequent flows compared to those recommended for a virtual consultation. For example, in our data, 66% (75,587/114,525) of users receiving an initial recommendation for an in-person ED visit initiated another flow compared to 9% (7241/80,451) of users recommended for a virtual consultation.

### Interpretation of Findings

Digital health technologies, particularly mHealth apps, are increasingly central to health care access, with virtual triage playing a crucial role in managing patient flow and clinical risk in urgent care settings [20-22]. Our finding that most (43,131/62,006) users navigated more than 1 self-triage flow suggests that the initial pathway or recommendation often did not fully align with the user’s immediate goal or expectation. While we could not definitively ascertain user motivations retrospectively, the observation that users receiving an ED referral were more likely to explore further flows supports the hypothesis that a primary driver for accessing alternative pathways may be a desire to find a route leading to a virtual consultation rather than an in-person visit. This aligns with literature suggesting patient preferences can be influenced by factors such as perceived condition severity, convenience, and avoidance of physical facility visits, even when algorithms flag potential risks [23].

The tendency to explore multiple flows, rather than strictly adhering to the first recommendation, highlights a complex interaction between algorithm design and user behavior. Patients might explore options, express uncertainty about their symptoms, seek reassurance, or attempt to bypass a recommendation perceived as inconvenient [24]. The finding that most flow interactions involved only 1 or 2 screens before cessation or switching suggests users may quickly identify when a pathway is heading toward an undesired outcome (such as an ED referral based on early red-flag questions) and promptly seek alternatives [16,25]. This rapid assessment, combined with the low average time per screen (approximately 9.5 seconds), points toward an efficient user interface and underscores the users’ proactive navigation to potentially achieve their desired outcome of a virtual visit.

The infrequent use of the back navigation feature (5368/62,006, 8% of users) is noteworthy. Instead of modifying answers within a single flow, users predominantly chose to initiate entirely new flows when dissatisfied or uncertain. This might suggest users perceive starting over with a different chief concern as a more effective strategy to find a suitable pathway within the app’s structure, rather than attempting to alter responses within a flow that already seems destined for an ED referral [14,26,27]. It could also simply reflect a user interface design where initiating a new flow is more intuitive or accessible than backtracking.

Claims regarding the specific demographic profile (eg, higher socioeconomic status) of typical symptom checker users found in some studies conducted elsewhere cannot be directly confirmed or refuted by our limited demographic data (age and gender only) [28-30]. However, the observed behaviors in our large cohort accessing the HIAE service, that is, seeking virtual care options even when directed elsewhere, suggest a strong inclination toward using mHealth solutions in this population.

This study contributes to the understanding of how users interact dynamically with self-triage interfaces beyond simple adherence rates. The efficiency suggested by the short interaction times is a positive indicator for system optimization, as minimizing waiting times is crucial for patient satisfaction. However, the prevalent flow-switching behavior highlights a potential mismatch between the algorithm’s safety-oriented design (high sensitivity for ED referrals) and the care preferences of a significant portion of users seeking virtual urgent care.

### Future Directions

To expand on our findings, future research should qualitatively investigate users’ motivations for navigating multiple flows through interviews or surveys to better understand the decision-making process behind flow switching. In addition, future studies that include formal usability assessments, such as the System Usability Scale or User Experience Questionnaires, could evaluate how the app’s user interface and user satisfaction influence navigation behavior. Exploring these questions in varied settings and with different symptom checker platforms will help determine if our findings apply beyond this single app. Such research could inform further improvements to self-triage systems, aiming to better balance safety protocols with user expectations and reduce the need for users to seek alternative pathways.

### Limitations

This study has several limitations. Its retrospective, single-center design, based in a private Brazilian health care setting, may limit the generalizability of findings to public health systems or different cultural contexts. The user population accessing this specific service may not represent the broader population. We relied on transactional log data, which provided valuable insights into navigation patterns but did not capture the users’ explicit reasoning, motivations, or satisfaction levels. Therefore, interpretations regarding intent (eg, avoiding ED visits) remain hypothetical, derived from observed behaviors. Furthermore, our analysis focused on interaction patterns and did not assess the clinical appropriateness of the triage recommendations or final health outcomes. The demographic data available for analysis was limited, precluding deeper investigation into how factors such as socioeconomic status or health literacy might influence interaction patterns. We also could not track users who abandoned the app entirely without completing at least 1 flow.

Our analysis was based on retrospective log data and did not include a direct user experience survey (eg, System Usability Scale or User Experience Questionnaire). This was a conscious scope decision, as our focus was on observed behavior at scale without involving additional participant input. We acknowledge that not capturing subjective usability feedback is a limitation, and as a result, we cannot directly determine whether user satisfaction or interface usability affected navigation behavior. We have now emphasized the importance of this aspect and suggest that future studies incorporate standardized usability tools alongside log analysis to assess how user experience might influence navigation patterns.

Finally, while the app was developed based on internal expertise, potential confirmation bias regarding its usability cannot be entirely ruled out, although we have attempted to present findings objectively.

### Conclusions

In this study, we investigated how patients navigate a symptom-based self-triage app in a DTC telemedicine setting. We found that only about one-third followed a single self-triage pathway as recommended. Most users explored alternative flows, particularly when the initial recommendation was for an in-person ED visit, while rarely using back navigation within a flow. This suggests a user preference for seeking virtual resolutions and highlights a dynamic interaction with triage algorithms.

In conclusion, our findings highlight areas for improvement in self-triage design. Future work should explore user motivations and include user experience metrics to improve digital triage tools and better meet patient needs.

## Data Availability

The datasets generated or analyzed during this study are available from the corresponding author upon reasonable request.

## Abbreviations

DTC: direct-to-consumer
ED: emergency department
HIAE: Hospital Israelita Albert Einstein
mHealth: mobile health
